# Optical clumped isotope thermometry of carbon dioxide

**DOI:** 10.1038/s41598-019-40750-z

**Published:** 2019-03-18

**Authors:** Ivan Prokhorov, Tobias Kluge, Christof Janssen

**Affiliations:** 1 Institute of Environmental Physics, Heidelberg University, 69120 Heidelberg, Germany; 2 Heidelberg Graduate School of Fundamental Physics, Heidelberg University, 69120 Heidelberg, Germany; 3 LERMA-IPSL, Sorbonne Université, CNRS, Observatoire de Paris, PSL Université, 75005 Paris, France

**Keywords:** Geochemistry, Geochemistry, Infrared spectroscopy

## Abstract

Simultaneous analysis of carbon dioxide isotopologues involved in the isotope exchange between the doubly substituted ^13^C^16^O^18^O molecule and ^12^C^16^O_2_ has become an exciting new tool for geochemical, atmospheric and paleoclimatic research with applications ranging from stratospheric chemistry to carbonate-based geothermometry studies. Full exploitation of this isotope proxy and thermometer is limited due to time consuming and costly analysis using mass spectrometric instrumentation. Here, we present an all optical clumped CO_2_ isotopologue thermometer with capability for rapid analysis and simplified sample preparation. The current development also provides the option for analysis of additional multiply-substituted isotopologues, such as ^12^C^18^O_2_. Since the instrument unambiguously measures all isotopologues of the ^12^C^16^O_2_ + ^13^C^16^O^18^O $\rightleftharpoons$ ^13^C^16^O_2_ + ^12^C^16^O^18^O exchange, its equilibrium constant and the corresponding temperature are measured directly. Being essentially independent of the isotope composition of the calibration gas, an uncalibrated working reference is sufficient and usage of international calibration standards is obsolete. Other isotopologues and molecules can be accessed using the methodology, opening up new avenues in isotope research. Here we demonstrate the high-precision performance of the instrument with first gas temperature measurements of carbon dioxide samples from geothermal sources.

## Introduction

Mass spectrometry of multiply substituted isotopologues or clumped isotopes has become an extremely powerful tool in the natural sciences. Demonstrated applications which investigated carbon dioxide, methane, nitrous oxide, molecular hydrogen and oxygen range from tectonic history and evolution, geobiology and atmospheric chemistry over the investigation of non-equilibrium processes with correction procedures, diagenesis studies, the investigation of mineral formation conditions, the assessment of hydrothermal flow systems and paleo-evolution to paleothermometry; and there are many more potential applications^1,2^. The most prominent uses are linked to the oxygen isotope exchange reaction between the main isotopologue and the ^13^C-^18^O containing species of CO_2_

R1 $${}^{12}{\rm{C}}{}^{16}{\rm{O}}_{2}+{}^{13}{\rm{C}}{}^{16}{\rm{O}}{}^{18}{\rm{O}}\rightleftharpoons {}^{13}{\rm{C}}{}^{16}{\rm{O}}_{2}+{}^{12}{\rm{C}}{}^{16}{\rm{O}}{}^{18}{\rm{O}}.$$

Reaction (R1) involves only a single chemical compound, but could not be exploited scientifically until recently. This is because their very low natural abundance hampers the study of multi-substituted isotopic molecules, such as ^13^C^16^O^18^O, containing two or more rare isotopes (e.g. ^13^C and ^18^O) simultaneously. When compared to the main isotopologue ^12^C^16^O_2_, these are well below 10^−4^ (see Table 1). At the same time, the measurement techniques needed to attain extremely high accuracy levels of a few 0.01‰ (~tens of ppm) in order to trace the natural variability of the corresponding isotopologue and a dynamic range on the order of about 10^9^ or better is therefore required. So far, only mass spectrometer instruments are capable of fulfilling these criteria and clumped CO_2_ has not yet been measured by optical methods^1,2^. Doubly substituted ^13^CH_3_D methane, which shows higher fractionation values, however, has been investigated using laser-based instruments. The first laser spectrometer setup^3^ for clumped methane isotopologues based on difference frequency generation (DFG) still suffered from uncertainties in the 20‰ range which exceeds natural ^13^CH_3_D variability of about 8‰^4^. Nevertheless, a more recent diode laser study on doubly substituted methane^5^ has successfully demonstrated that optical systems can well approach the necessary requirements. The achieved precision level of 200 ppm, however, remains still well above the commonly accepted threshold of 100 ppm (or 0.1‰) required for the study of clumped isotope fractionation in non-hydrogenated molecules.

**Table 1:** Typical relative abundance of stable CO_2_ isotopologues in decreasing order.

Isotopologue i		Mass (u)^a^	Rel. abundance *n*_i_/Σ_j_*n*_j_ (mol/mol)	Rel. contribution to mass (%)	Note
No	Symbol				
1	^12^C^16^O_2_	44	9.842 ⋅ 10^−1^	100.000	
2	^13^C^16^O_2_	45	1.100 ⋅ 10^−2^	93.636	
3	^12^C^16^O^18^O	46	3.947 ⋅ 10^−3^	99.785	
4	^12^C^16^O^17^O	45	7.478 ⋅ 10^−4^	6.364	b
5	^13^C^16^O^18^O	47	4.413 ⋅ 10^−5^	96.710	
6	^13^C^16^O^17^O	46	8.361 ⋅ 10^−6^	0.211	
7	^12^C^18^O_2_	48	3.957 ⋅ 10^−6^	99.578	c
8	^12^C^17^O^18^O	47	1.500 ⋅ 10^−6^	3.286	
9	^12^C^17^O_2_	46	1.421 ⋅ 10^−7^	0.004	
10	^13^C^18^O_2_	49	4.424 ⋅ 10^−8^	100.000	
11	^13^C^17^O^18^O	48	1.676 ⋅ 10^−8^	0.422	
12	^13^C^17^O_2_	47	1.588 ⋅ 10^−9^	0.003	

Abundance values are based on assuming a statistical distribution of oxygen and carbon isotopes in international standard materials (VSMOW for O and VPDB for C: ^13^*R* = 11056/988944, ^17^*R* = 3790/9976206, ^18^*R* = 20004/9976206)^53^. ^a^Atomic mass constant. ^b^Can only be measured after conversion^10,11^ into O_2_ or using isotope exchange techniques^12^. ^c^Signal used to detect contaminant species, such as hydrocarbons or halogenated compounds^14^.

While mass spectrometers excel in the achieved precision of about 10 to 20 ppm^6,7^, the instruments have to cope with inherent drawbacks. Not only are they relatively costly and heavy, thus not permitting in-field operation; they also require time consuming measurements and careful sample preparation in order to avoid contamination of the measurement signal. With current mass spectrometric procedures, preparation and analysis of a carbonate sample take about 3 to 6 h^8^. Importantly, only the largest and most sophisticated instruments can reach the mass resolution required to resolve isobaric interferences in CO_2_^9^. Typical operation conditions are around *M*/*ΔM* ~ 40000 or lower, which is insufficient to separate ^13^C^16^O_2_ from ^12^C^16^O^17^O at m/z = 45 or ^13^C^16^O^18^O from ^12^C^17^O^18^O at m/z = 47, for example. In order to resolve these masses, a resolving power of above 52000 is needed – so far only accessible for large radius instruments in ‘non-normal operation’ mode^9^. This makes multiply substituted isotopologue analysis by mass spectrometry a very exclusive technology that will remain limited to only a handful of highly specialised laboratories worldwide, likely also constraining industrial or commercial use. Note that only two (^12^C^16^O_2_ and ^13^C^18^O_2_) out of twelve stable CO_2_ isotopologues can be detected entirely free from isobaric interference using a mass spectrometer (see Table 1). The four isotopologues ^13^C^16^O_2_, ^12^C^16^O^18^O, ^13^C^16^O^18^O, and ^12^C^18^O_2_, strongly dominate (>90%) a cardinal mass signal. They can thus well be assessed by the same technology except ^12^C^18^O_2_, whose quantification suffers from distorting background signals. For minor contributions to a cardinal mass, such as ^12^C^16^O^17^O however, more advanced sample preparation or conversion technologies^10–12^ must be employed at the cost of prolonged measurement time and reduced precision.

Simple counting statistics prevent using current mass spectrometer technology for the analysis of CO_2_ isotopologues below the relative abundance level of 10^−5^, even if these provide the main contribution to a cardinal mass. Assuming the measurement uncertainty being limited by Poisson statistics, the 10 ppm precision is reached after about 3 or 4 h of measurement on m/z = 47 (^13^C^16^O^18^O)^13^. In order to obtain the same precision for the ^12^C^18^O_2_ isotopologue on m/z = 48, a (*n*(^16^ ^13^C^16^O^18^O)/*n*(^12^C^18^O_2_))^2^ ~ 100 times longer analysis time would be required, thus about two weeks. Even measurement times of two days are impractical and contrary to common practice. Demonstrations of ppm level instrument stabilities over such long time periods are lacking too. The m/z = 48 and 49 signals can therefore only be used as an indicator for sample contamination (hydrocarbons, halocarbons, sulphur monoxide)^1,14,15^ and must remain useless in exploiting ^12^C^18^O_2_ or ^13^C^18^O_2_ as isotopic tracers with mass spectrometry.

Despite the pioneering achievements of mass spectrometry in rare multi-isotope research, it is evident that alternative technologies are needed to overcome several of the aforementioned limitations. In this paper, we will present the first optical multi-isotopologue analyser for CO_2_ that is not influenced by most of these limitations, most notably the isobaric interference problem. The instrument achieves a measurement accuracy well below the 100 ppm level in the measurement of ^13^C^16^O^18^O and the technique has the capacity of assessing new tracers such as ^12^C^18^O_2_ that likely provide new and complementary information. In principle the developed method is calibration free and has strong potential of becoming a breakthrough technology, because it provides great isotopologue selectivity at reduced time, size and cost factors, which makes it well suited for widespread scientific, laboratory and commercial applications. The paper focusses on the measurement of carbon dioxide and the isotope exchange in the gas phase and we present the analysis of CO_2_ from thermal sources in the Upper Rhine Valley. The results will be compared to duplicate mass spectrometer measurements of CO_2_ from the same source. Apart from direct studies of gaseous carbon dioxide, current applications are concerned with multiply-substituted isotopologues in carbonates. Because carbonate isotopologues are obtained from measurements of gaseous CO_2_ released during the acid digestion of the carbonates, they can be investigated using the same analysis systems. The direct application of carbon dioxide isotopologue analysis to carbonates is further facilitated by the fact that the carbonate clumped isotope scale has been directly tied into the equilibrium CO_2_ gas scale^6^.

## Operating Principles and Instrumental Approach

### Isotopologue absorption spectroscopy

**Figure 1:**
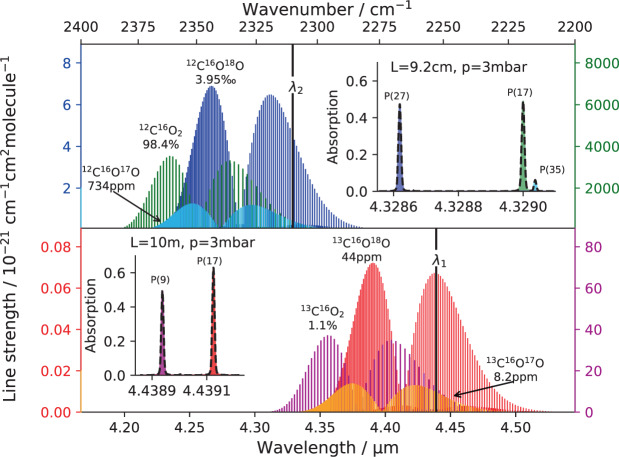
Mid-infrared spectra of CO_2_ isotopologues. The top panel shows spectra around the 4.33 µm laser wavelength for the detection of the main isotopologue (^12^C^16^O_2_), and the two singly substituted oxygen species ^12^C^16^O^17^O and ^12^C^16^O^18^O. The lower panel displays spectra of ^13^C containing isotopologues (^13^C^16^O_2_, ^13^C^16^O^17^O, and ^13^C^16^O^18^O) at 4.44 µm. Broadband spectra in main panels were simulated using the HITRAN^41^ database. The insets zoom in on the measured absorption of selected lines at corresponding path lengths and the typical sample pressure of 3 mbar. Black dashed lines represent the experimentally measured spectra and shaded areas the fit of the respective isotopologue line area.

**Figure 2:**
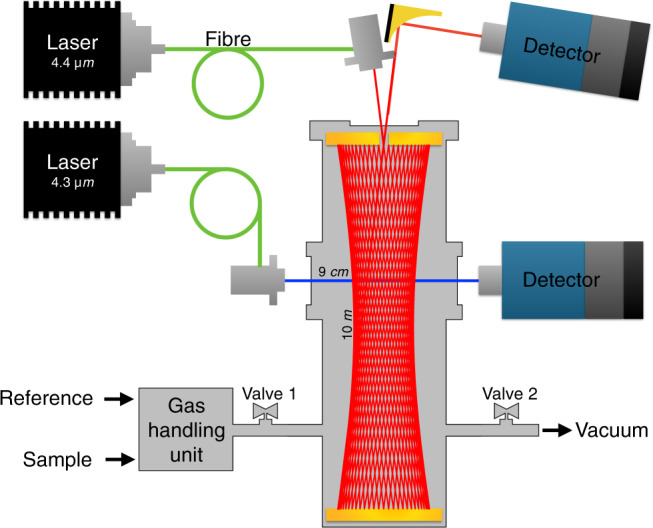
Scheme of the dual-laser system. Lasers are connected to the absorption cell via optical fibres. An off-axis parabolic mirror focusses the exiting light from the multi-pass cell on a photo-detector. The light of the single pass is projected on a second detector without further focussing. The cell is filled with sample and reference gases via a custom-built inlet system and can be evacuated using a second gas connection.

The optical measurement of CO_2_ isotopologues is based on absorption of isotopologue dependent ro-vibrational transitions in the *ν*_3_ fundamental band around 4 µm (Fig. 1), where infra-red absorption of carbon dioxide is strongest. With respect to the ^12^C containing species, the vibrational bands of ^13^C containing isotopologues are shifted towards lower energies. Using simultaneously two tuneable inter-band cascade lasers (ICLs) at 4.3 and 4.4 µm and absorption-balanced light paths of 9.2 cm and 10 m length, respectively, absorption signals of several ten percent of the five most abundant isotopologues can be obtained when a few mbar of pure CO_2_ are analysed in a cylindrical 0.8 L thermostated stainless steel absorption cell. A Herriott cell configuration made out of two concave mirrors at a distance of about 17.6 cm has been realised in order to achieve the long light path with 58 reflections. The short path traverses the cell just once in the perpendicular direction. More information on the absorption lines and a more detailed description of the set up displayed in Fig. 2 are given elsewhere^16^.

Spectra of pure CO_2_ at 297 K and 3 to 4 mbar are recorded at a rate of 1.56 kHz by driving laser currents at the same pace. A non-linear current ramp has been chosen for minimising the non-linearity of the laser frequency response over time^17^. Individual spectra were then averaged over 1 s time interval and analysed using a home-built fitting routine employing Rautian line profiles^18^. Required line parameters have been taken either from the HITRAN database (position) or were determined in separate experiments (self broadening coefficient and frequency of velocity changing collisions)^16^. The particle number density *n* of an isotopologue is obtained from applying the Beer-Lambert law to one of its transitions (see insets in Fig. 1), located at the centre wavenumber *ν* = *ν*_*c*_. For a spectrally narrow laser the CO_2_ gas number density (*n*(CO_2_) = [CO_2_]) is linked to the optical measurement via^19^

1 $$[{{\rm{CO}}}_{2}]=-\frac{\mathrm{ln}(Tr(\nu ))}{S\cdot g(\nu -{\nu }_{c})\cdot L}=\frac{\alpha }{\sigma \cdot L},$$

where *L* is the path length, *Tr*(*ν*) the transmittance, *S* the line strength and *g*(*ν* − *ν*_*c*_) the molecular line shape function of the particular transition. Isotopologue concentrations can thus be obtained from the extinction coefficient *α* = −ln(*Tr*(*ν*_*c*_)) and the absorption cross section *σ* = *S* ⋅ *g*(0) at peak centre, which remain constant under fixed experimental conditions. The path length cancels in the measurement of an isotopologue ratio when both isotopologues are detected using the same path, e.g.^20^. [CO_2_]_1_/[CO_2_]_2_ = *α*_1_/*α*_2_⋅(*σ*_1_/*σ*_2_)^−1^, making the method immune against eventual slight path length changes. Note that in spite of using the simplifying notation on the right hand side of Eq. (1) spectra are fitted over a whole frequency window and therefore include information from entire absorption lines and not just from peak absorption values.

Duplicate isotope ratio mass spectrometer (IRMS) measurements have been performed using the ThermoFischer MAT253Plus instrument at IUP Heidelberg, that has been equipped with an additional m/z = 47.5 cup and 10^13^ Ω resistors on m/z = 47−49 mass cups^16^. The m/z = 47.5 is used for continuous baseline monitoring. The mass spectrometric analysis follows accepted procedures^6,14^, as does the sample processing and cleaning^8^.

### Equilibrium constant, thermodynamics and Δ_47_

**Figure 3:**
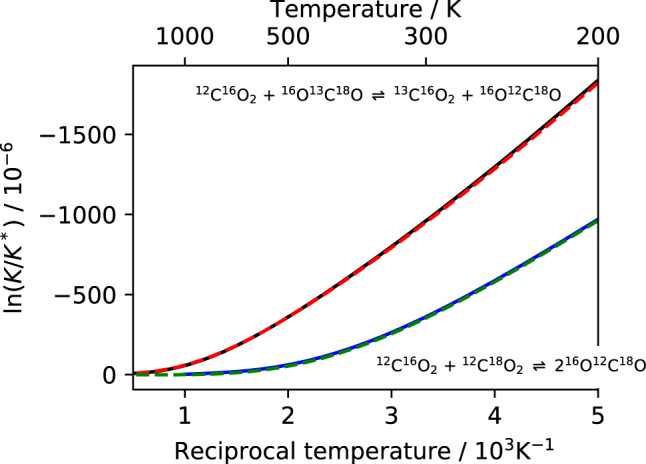
Isotope equilibria of the ^12^C^16^O_2_ + ^13^C^16^O^18^O (R1) and the ^12^C^18^O_2_ + ^12^C^16^O_2_ (R2) exchange reaction at temperatures between 200 and 1500 K. The logarithm of the normalised equilibrium constants $K/{K}^{\ast }$ is shown, where ${K}_{1}^{\ast }=1$ and ${K}_{2}^{\ast }=4$ are the statistical (high temperature) limits. Statistical mechanics calculations^22^ from partition function ratios according to Bigeleisen-Mayer-Urey (BMU, hereafter) theory^25^ (red and green dashes) and direct sum calculations (black and blue lines) based on new spectroscopic data generated from experimentally refined ab initio calculations^34^ are displayed.

The equilibrium constant *K*_1_ of the isotope exchange reaction (R1) is strictly proportional to the product of absorption signals $A={\alpha }_{{}^{13}{\rm{C}}{}^{16}{\rm{O}}_{2}}\,{\alpha }_{{}^{12}{\rm{C}}{}^{16}{\rm{O}}{}^{18}{\rm{O}}}\,{\alpha }_{{}^{12}{\rm{C}}{}^{16}{\rm{O}}_{2}}^{-1}\,{\alpha }_{{}^{13}{\rm{C}}{}^{16}{\rm{O}}{}^{18}{\rm{O}}}^{-1}$, which therefore allows for the optical measurement via

2 $${K}_{1}=\frac{[{}^{13}{\rm{C}}{}^{16}{{\rm{O}}}_{2}][{}^{12}{\rm{C}}{}^{16}{\rm{O}}{}^{18}{\rm{O}}]}{[{}^{12}{\rm{C}}{}^{16}{{\rm{O}}}_{2}][{}^{13}{\rm{C}}{}^{16}{\rm{O}}{}^{18}{\rm{O}}]}=\Sigma \times A,$$

where the scaling factor $\Sigma ={\sigma }_{{}^{12}{\rm{C}}{}^{16}{\rm{O}}_{2}}\,{\sigma }_{{}^{13}{\rm{C}}{}^{16}{\rm{O}}{}^{18}{\rm{O}}}\,{\sigma }_{{}^{13}{\rm{C}}{}^{16}{\rm{O}}_{2}}^{-1}\,{\sigma }_{{}^{12}{\rm{C}}{}^{16}{\rm{O}}{}^{18}{\rm{O}}}^{-1}$ depends on the involved molecular line strengths. Lacking the required accuracy, current database values cannot be used for determining Σ, which best is determined experimentally by exploiting the temperature dependence of *K*_1_ or its logarithm (see Fig. 3). The latter is widely used, because deviations from the statistical value $({K}_{1}^{\ast }=1$, where the *-symbol indicates the high temperature limit) are small and it can be expressed by three individual logarithmic terms, which can be identified with the isotopologue specific enrichment or fractionation values (CO_2_ denoting any particular isotopologue in the following equation)

3 $$\Delta ({{\rm{CO}}}_{2})=\frac{[{{\rm{CO}}}_{2}]}{{[{{\rm{CO}}}_{2}]}^{\ast }}/\frac{[{}^{12}{\rm{C}}{}^{16}{\rm{O}}_{2}]}{{[{}^{12}{\rm{C}}{}^{16}{\rm{O}}_{2}]}^{\ast }}-1\simeq \,\mathrm{ln}\,\mathrm{(1}+\Delta ({{\rm{CO}}}_{2})),$$

commonly used for the quantification of isotopomers^21^ or multiply substituted isotopologues^22,23^:

4 $$-\,\mathrm{ln}(\frac{{K}_{1}}{{K}_{1}^{\ast }})=\,\mathrm{ln}(\frac{[{}^{13}{\rm{C}}{}^{16}{\rm{O}}{}^{18}{\rm{O}}]}{[{}^{13}{\rm{C}}{}^{16}{\rm{O}}_{2}]}\frac{[{}^{12}{\rm{C}}{}^{16}{\rm{O}}_{2}]}{[{}^{12}{\rm{C}}{}^{16}{\rm{O}}{}^{18}{\rm{O}}]})=\,\mathrm{ln}(\frac{[{}^{13}{\rm{C}}{}^{16}{\rm{O}}{}^{18}{\rm{O}}]}{[{}^{13}{\rm{C}}{}^{16}{\rm{O}}_{2}]})-\,\mathrm{ln}(\frac{[{}^{12}{\rm{C}}{}^{16}{\rm{O}}{}^{18}{\rm{O}}]}{[{}^{12}{\rm{C}}{}^{16}{\rm{O}}_{2}]})\mathrm{.}$$

The right hand side expression is applicable to the optical measurement, which provides the two particular isotopologue ratios as independent observables. As evident from Eq. (4), the temperature information contained in the equilibrium constant does only depend on two concentration ratios, that may be regrouped differently. ln (*K*_1_) is completely independent of the bulk isotope composition. Consequently, the ^17^O isotopic composition does not at all affect the determination of the equilibrium constant. Bulk isotopic compositions are only introduced when isotopologue concentrations are replaced by Δ values as defined in Eq. (3). Using $${K}_{1}^{\ast }=1={[{}^{12}{\rm{C}}{}^{16}{\rm{O}}{}^{18}{\rm{O}}]}^{\ast }{[{}^{13}{\rm{C}}{}^{16}{\rm{O}}_{2}]}^{\ast }/$$$$({[{}^{12}{\rm{C}}{}^{16}{\rm{O}}_{2}]}^{\ast }{[{}^{13}{\rm{C}}{}^{16}{\rm{O}}{}^{18}{\rm{O}}]}^{\ast })$$ and keeping only the leading terms in the Taylor series expansion on both sides, one obtains

5 $$\frac{{K}_{1}}{{K}_{1}^{\ast }}-1\simeq \Delta ({}^{13}{\rm{C}}{}^{16}{\rm{O}}{}^{18}{\rm{O}})-\Delta ({}^{13}{\rm{C}}{}^{16}{\rm{O}}_{2})-\Delta ({}^{12}{\rm{C}}{}^{16}{\rm{O}}{}^{18}{\rm{O}}),$$

where the $\simeq$ sign indicates the approximative character of the relation. This equation, which may also be derived directly from Eq. (24) of Wang *et al*.^22^, is very similar to the commonly used definition of Δ_47_ in clumped isotope mass spectrometry of CO_2_ ^14,24^,

6 $${\Delta }_{47}=\Delta ({}^{47}{\rm{C}}{{\rm{O}}}_{2})-\Delta ({}^{46}{\rm{C}}{{\rm{O}}}_{2})-\Delta ({}^{45}{\rm{C}}{{\rm{O}}}_{2}),$$

where isotopologues are replaced by m/z signals, because they cannot be measured individually. Comparison of Eqs (5) and (6) leads to the identification of ${\Delta }_{47}\simeq {K}_{1}/{K}_{1}^{\ast }-1$ with the relative deviation of the equilibrium constant *K*_1_ from its statistical value. It has been argued that the last two terms on the right hand side are zero^24^, but this premise is not completely consistent with the definition of Δ in Eq. (3) and thermodynamic calculations^22^ that respectively yield −4 and −11 ppm for Δ(^45^CO_2_) and Δ(^46^CO_2_) for CO_2_ equilibrated at 300 K. The reason for the conflicting results is that in the one case approximate but precisely measurable and in the other case exact but only approximatively accessible atomic isotope ratios are used in the calculations of the statistical abundances. Nonetheless, the so defined Δ_47_ is overwhelmingly influenced by the first of the three terms, which in turn is to a large extent (97%, see Table 1) dominated by the ^13^C^16^O^18^O isotopologue.

Unlike the direct measurement of ln (*K*_1_) according to Eq. (4), mass spectrometer determinations of Δ_47_ not only require measurement of heavy isotopologue abundances. The ‘absolute’ or bulk isotope composition must also be known in order to determine the statistical abundance of the m/z = 47 signal. This implies determining atomic ^13^C/^12^C, ^18^O/^16^O or ^17^O/^16^O ratios (traditionally quantified in terms of *δ*^13^C, *δ*^18^O and *δ*^17^O values), necessitating that international standard substances are used and that assumptions on the ^17^O isotope content are made. In this way systematic biases of up to 40 ppm are introduced^24^. Equally important, mass spectrometers can only approximately access the clumped ^13^C^16^O^18^O isotopologue (also due to an ion-source dependent scrambling effect) using the m/z = 47 signal and a corresponding scaling factor must be applied^1,14^.

Using the equilibrium constant of an isotope exchange (or isomerisation) reaction with a particular working gas as a thermometer, temperature is directly measured as a thermodynamic variable. The equilibrium constant of an isotope exchange reaction is linked to the reaction free enthalpy Δ*F* of that reaction *K* = exp(−Δ*F*/(*kT*)) ^25–27^, where *k* is the Boltzmann constant and where we have adopted a per molecule rather than a per mole definition of energies. The free energy of the gas is linked to the gas’ molecular partition function, which sums over all energy states *ε*_*i*_ taking degeneracies *d*_*i*_ into account and counting internal energy states from the lowest or zero-point energy (ZPE)  *ε*_0_ state of the molecule

7 $$Q=\mathop{\sum }\limits_{i}^{levels}\,{d}_{i}{e}^{-\frac{{\varepsilon }_{i}}{kT}}={Q}_{trans}{Q}_{int}{e}^{-\frac{{\varepsilon }_{0}}{kT}}\mathrm{.}$$

In the Eq. (7) we have made the usual separation of the centre of mass motion (*trans*) from the molecular internal degrees of freedom (*int*). Since the equilibrium constant is given as a product of partition functions of reactant (*react*) and product (*prod*) molecules^26,27^

8 $$K=\frac{\prod \,{Q}_{prod}}{\prod \,{Q}_{react}}={(\frac{\prod {M}_{prod}}{\prod {M}_{react}})}^{\mathrm{3/2}}\frac{\prod \,{Q}_{int,prod}}{\prod \,{Q}_{int,react}}\exp (-\frac{\sum \,{\varepsilon }_{\mathrm{0,}prod}-\sum \,{\varepsilon }_{\mathrm{0,}react}}{kT}),$$

it is amenable to quantum statistical mechanics and computational chemistry methods. Here we have followed the usual simplification to evaluate the ratio of translational partition functions to ratios of molecular masses *M*^26^. The only non-trivial factors are the total internal partition functions that need to be evaluated separately. If one is mainly interested in isotope fractionation effects, it is convenient to normalise the equilibrium constant by dividing through its classical high temperature limiting value K*, which is given as the product of the classical symmetry numbers of product and reactant molecules:

$$\frac{K}{{K}^{\ast }}=K\frac{\prod \,{\sigma }_{react}}{\prod \,{\sigma }_{prod}}.$$

Solving for $\Delta {\varepsilon }_{0}=\sum \,{\varepsilon }_{\mathrm{0,}prod}-\sum \,{\varepsilon }_{\mathrm{0,}react}$, one obtains the ZPE change of the reaction in terms of measurable quantities:

9 $$\Delta {\varepsilon }_{0}=-\,kT[\mathrm{ln}\,K-\frac{3}{2}\,\mathrm{ln}(\frac{\prod \,{M}_{prod}}{\prod \,{M}_{react}})-\,\mathrm{ln}(\frac{\prod \,{Q}_{int,prod}}{\prod \,{Q}_{int,react}})].$$

The different terms in Eq. (9) can be identified with the reaction enthalpy or energy Δ*H* = Δ*U* = Δ*ε*_0_, the reaction free enthalpy ($\Delta F=-\,kT\,\mathrm{ln}\,K$), and the free enthalpy change associated to the reaction entropy (*T*Δ*S*), which is given by the two remaining terms on the right hand side. Eq. (9) takes into account the energy change associated with the translational and internal molecular motion. In the following we adopt spectroscopic conventions and use term energies in wavenumber units Δ*ν* = Δ*ε*/*hc*, where *h* and *c* are the Planck constant and the speed of light, respectively. Different methods have been proposed to calculate the internal partition functions in Eq. (7). The traditional method based on work of Urey, Bigeleisen and Goeppert-Mayer (BMU)^25–27^ is to consider molecules as rigid rotor – harmonic oscillators and use corresponding spectroscopic constants. Combination of this approach with the Teller-Redlich rule^28^, usually attributed to Urey^29^, leads to a very simple description. For better accuracy, anharmonic corrections to vibrational energies and rotation-vibration interactions can be taken into account^25,30,31^, but for reasons of convenience or lack of parameters mostly only the anharmonic corrections to the ZPE are applied^22,29^. This can lead to significant uncertainties and more elaborate methods have been proposed, such as calculating the direct sum as a path integral using Monte-Carlo (PIMC) methods^32,33^. If highly accurate potential surfaces with spectroscopic quality are available or global effective Hamiltonians have been determined, such as for CO_2_ ^34^, the total internal partition function in Eq. (7) might also be calculated very accurately by summing directly over all terms. In any case, the standard BMU approach must fail at low temperatures and masses (e.g. H_2_) due to neglecting the quantisation of rotational states, which is only taken into account approximately. In such a case the sum in Eq. (7) must be evaluated directly^25^. Conversely, the computational cost associated with numerical methods, such as PIMC and direct summation will increase when the temperature augments, because the number of thermally accessible states increases strongly. In addition the potential energy surface properties far from the equilibrium configuration become important when temperatures raise, leading to numerical convergence problems and artefacts^22,35^. In this limiting case, where isotope fractionation must vanish and a high precision is required, the BMU approach in combination with the Teller-Redlich rule might serve as a particularly useful guide, because its convergence towards the statistical limit is always assured.

### Implementation of the spectroscopic measurement

Unlike mass spectrometry, a laser absorption instrument can unambiguously measure all four (or three) required isotopologues of a homogenous CO_2_ isotope exchange reaction. The measurement is conceptually straight forward and does not depend on additional determinations and hypotheses on the bulk isotopic composition, as it directly determines ln *K* or $\mathrm{ln}\,(K/{K}^{\ast })$ and its temperature dependence, disregarding the ln Σ term (see Eqs (2) and (4)), which needs to be determined experimentally using a reference measurement:

10 $${\rm{l}}{\rm{n}}\,{K}_{1}(T)=\,{\rm{l}}{\rm{n}}\,A(T)+\,{\rm{l}}{\rm{n}}\,\Sigma =\,{\rm{l}}{\rm{n}}\,{K}_{1}({T}_{ref})+\,{\rm{l}}{\rm{n}}(A(T)/{A}_{ref}).$$

Here, *A*(*T*) and *A*_*ref*_ indicate the measured product of absorbances in Eq. (2) for CO_2_, once for the sample and once for a reference gas with known equilibrium constant *K*_1_ that has been equilibrated at the reference temperature *T*_*ref*_. Consequently, the method makes the quantification of the statistical distribution of isotopes obsolete (as indicated by Eq. (4)). Since absolute abundances of C and O isotopes don’t need to be known, the absorption measurement dispenses in principle the use and measurement of international standard substances. It only requires the comparison with a working gas whose value of ln *K* is known and remains stable over time. The extremely slow gas phase isotope exchange at ambient temperatures assures that any equilibrated CO_2_ gas with an isotopic composition close to natural sample gas composition in principle suffices for determining ln Σ (see Eq. (10)). This should make laser-based clumped isotopologue analysis much easier applicable than mass spectrometer investigations. In our setup, however, a slight cross-sensitivity of ln *K* on the difference in *δ*^13^C between the sample and the working reference gas of −4 ppm/‰ has been observed. This entails the determination of *δ*^13^C in our samples such that the interference can be corrected empirically^16^. The correction requires only one extra measurement and evaluation step relative to the sophisticated and error-prone mass spectrometric procedure. On the contrary, a cross correlation between ln *K* and *δ*^18^O has not been observed. The *δ*^13^C interference is much stronger than the isotope dependence of the thermodynamic equilibrium composition of about −0.01 ppm/‰. The effect is likely due to an insufficient modelling of the baseline originating from strong nearby absorption features of ^13^CO_2_. An improved fitting algorithm and a better choice of the spectral micro-window for the absorption lines should eliminate the effect, but this hypothesis requires further examination.

A natural candidate for the calibration of the optical method using Eq. (10) are measurements of the equilibrium constant at the high temperature limit $(\mathrm{ln}\,{K}_{1}(T > 2000\,{\rm{K}})\simeq \,\mathrm{ln}\,{K}_{1}^{\ast }=0)$. As these conditions are difficult to realise experimentally, we use a two step calibration, involving a heated working reference gas at a lower temperature and an ambient temperature working reference gas. The hot CO_2_ serves as calibration point, that determines the origin of the optical ln *K*_1_ measurements. The gas has been equilibrated at 1000 °C for about 5 h. We use the calculated value of $\mathrm{ln}\,{K}_{1}=-\,26$ ppm at that temperature (see Table 2) to determine *A*_*ref*_ in Eq. (10). The uncertainty of this calibration is very small: different calculations at 1000 K are given in the literature^22,36^ and our calculation based on partition functions evaluated as direct sums of energy levels provided by ab initio calculations that were refined by spectroscopic measurements^34^, indicate that the error at that temperature is about 5 ppm. This deviates by only 2 ppm from the BMU method using the molecular constants of Wang *et al*.^22^. If we assume that the relative uncertainty remains the same, the systematic bias of the calibration should only be about 2 to 3 ppm at 1000 °C. Calculated room temperature (300 K) values of ln (*K*_1_) show a larger spread between −951 and −968 ppm, giving an order of ±10 ppm agreement (see Table 2). This uncertainty span is significantly larger than the spread in the high temperature values, rendering high temperature measurement preferable for calibration. As does the temperature gradient, which is 82 times smaller than the room temperature gradient of $d\,\mathrm{ln}\,{K}_{1}/dT=5.7$ ppm/K and makes the high temperature calibration less sensitive to instabilities in the temperature than its room temperature counterpart. This first calibration led us assign a value of ${\rm{l}}{\rm{n}}\,{K}_{1,ref}=-(954\pm 20)$ ppm to our room temperature reference gas (lab grade purity N4.8 from Air Liquide, *δ*^13^C_VPDB_ = −(40.0 ± 0.3)‰, ${\delta }^{18}{{\rm{O}}}_{{\rm{VPDB}}-{{\rm{CO}}}_{{\rm{2}}}}=-\,\mathrm{(27.3}\pm \mathrm{0.3)}$‰). If not noted otherwise, we indicate measurement uncertainties as combined standard uncertainties at the 68% level of confidence.

**Table 2:** Temperature dependence of the isotope equilibrium constant *K*_1_.

Temperature *T* (K)	−(ln *K*_1_)/10^−6^				
	WSE2004^a^ ^22^,	CL2012^54^	WM2014^33^	CBRZ2014^36^	This work
100	4830				4850
200	1827			1842	1836
273.15	1112				1122
300	954	951	960 (9)	968	959
400	566		570 (9)		570
500	358		357 (7)		359
600	235		232 (5)		235
1000	59			51	56
1273.15	28				26
1500	17				15
2000	7				8

Different theories are employed: BMU approach using harmonic frequencies for application of the Teller-Redlich^28^ rule and anharmonic correction to the ZPEs – WSE2004^22^. The same approach using harmonic frequencies from another level of theory – CL2012^54^. Path-Integral Monte Carlo (PIMC) evaluation of partition sums – WM2014^33^. Approximate direct sum partition functions from a refined potential surface – CBRZ2014^36^. Direct sum calculation of partition functions (this work) using state energies from new spectroscopic data generated from experimentally refined ab initio calculations^34^. ^a^Values at temperatures other than 200, 300 and 1000 K were recalculated from molecular constants in Table 3 of Wang *et al.*^22^.

In the second step, individual samples are measured by alternating acquisition sequences of sample and working gas. Each sequence starts by filling the spectrometer with the working reference gas and acquiring spectra for about 30 s with 1 s integration time. Then fast (~2 min) removal of the working reference occurs and the sample gas is analysed following the same acquisition procedure. Pressures of sample and reference gases are matched to agree within 0.01%. For repeated analysis, the sample is recovered using cryogenic trapping for about 5 minutes. At the end of the evacuation period the optical base line is determined. These repeated sample-reference comparisons, where Eq. (10) is applied each time, allow to take into account slow instrumental drifts that may have an impact on the determination of ln Σ.

## Results and Discussion

### CO_2_ thermometry with ^13^C^16^O^18^O and case application

**Figure 4:**
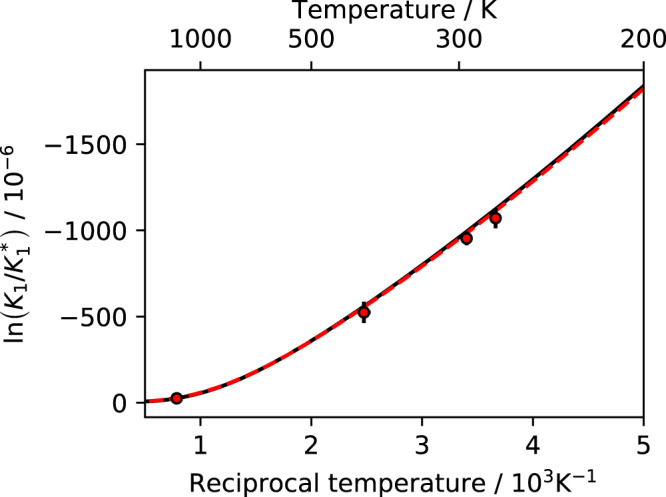
Measurement of the ^12^C^16^O_2_ + ^13^C^16^O^18^O equilibrium constant over the 274 to 1273 K (1 to 1000 °C) temperature range. ln (*K*_1_) is determined directly for samples of different isotopic composition that were equilibrated in quartz (*T* > 1000 K) or in pyrex tubes to which drops of liquid water were added (*T* = 274, 294 and 404 K). Data are given with combined standard uncertainties that include the uncertainty of the calibration procedure based on the working gas measurement. Solid and dashed lines indicate two different theoretical temperature dependencies (see text and caption Fig. 3 for more details). The high temperature value (practically error free) has been used to determine the isotopic composition of the room temperature working gas, with respect to which values at 274, 294 and 404 K have been determined.

The newly developed laser instrument has first been employed to demonstrate its capacity as CO_2_ isotopologue thermometer using ln *K*_1_ as directly observable temperature proxy. Four different samples of equilibrated CO_2_ have been prepared, with equilibration temperatures at 1 °C, 21 °C, 131 °C, and 1000 °C. For measurements at 1000 °C, pure CO_2_ gas was filled into quartz vials and kept in a lab oven for about 5 h. For the lower temperatures, droplets of liquid water were added to facilitate isotope exchange between isotopologues of CO_2_. At 1 °C equilibration times were about one month, and they were about a week for the intermediate temperature at 131 °C. Figure 4 shows the results of the measurements in comparison to the theoretically calculated curve. As a reference we use our evaluations of *K*_1_ from partition functions determined as direct sums and from the BMU method with harmonic frequencies and ZPE values given by Wang *et al*.^22^ (see Table 2). The maximum deviation of 59 ppm between either of the theoretical calculations in Fig. 4 and the measurements has been observed at 274 K. It is within twice the combined standard uncertainty (61 ppm) of the laser spectroscopic measurements at that temperature. At room temperature or above, the observed agreement is well within one standard uncertainty, which is 25 and 33 ppm, respectively.

**Figure 5:**
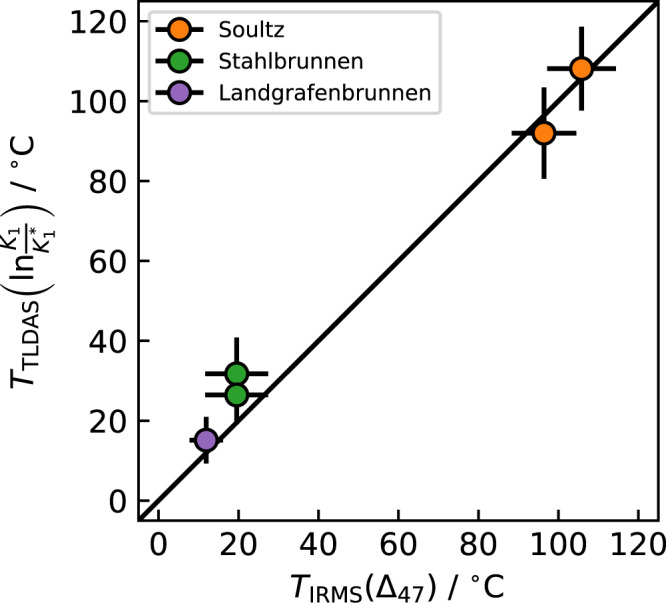
Comparison of optical (*y*-axis) and mass spectrometer (*x*-axis) measurements using natural samples from three sources in Germany and France. Two low temperature sources Landgrafenbrunnen (water temperature *T*_w_ = 13.5 °C) and Stahlbrunnen (water temperature *T*_w_ = 12.4 °C) are situated in Bad Homburg (50°13′26″ N, 8′37′21″ W) and are compared to a thermal source (sampling water temperature *T*_w_ = 38.5 °C) in Soultz (47°53′12″ N, 7°13′47″ W), France. Note that both clumped isotope equilibrium methods are consistent in magnitude and relative to each other. Uncertainties as well as combined preparation plus analysis times are similar for both, mass spectrometer and laser measurements.

Clumped isotope thermometry of gas phase CO_2_ originating from hydrothermal systems might provide a new and unique tracer for hydrothermal reservoir temperatures. The application is particularly relevant for studying the feasibility of the construction of hydrothermal power plants and estimating the associated risks, because the isotope thermometer may provide additional information on the related geological system and involved aquifers. For this case study we compare tuneable laser direct absorption spectroscopy (TLDAS) and IRMS measurements of the ^13^C^16^O^18^O isotopologue in a case study of natural carbon dioxide extracted from an operating hydrothermal power plant (Soultz) and two shallow wells (Landgrafenbrunnen and Stahlbrunnen), all located in the Upper Rhine Valley. The geothermal reservoir in Soultz (Alsace, France) has a temperature of about 200 °C at a depth of 5000 m^37^. During power plant operation, the water cools down to ~150 °C at the surface. Carbon dioxide for the analysis has been sampled from a separate sampling line, where the water has been rapidly cooled down to 38.5 °C. The mass spectrometric and laser measurements show clumped isotope temperatures between 92 and 108 °C and the two methods agree well within the respective uncertainties of 8 °C for the mass spectrometer determinations and 11 °C for the laser measurements (Fig. 5). Stahlbrunnen and Landgrafenbrunnen are two hydrothermal wells in Bad Homburg, Germany. The CO_2_ from the first one has been sampled directly from the well in the gas phase, whereas sampling of the carbon dioxide dissolved in water has been performed for the latter. The preparation of the gas samples for laser spectroscopic analysis follows a simplified procedure. It involves cryogenic separation from water and removal of non-condensable species through vacuum pumping. Compared to preparation for IRMS analysis, which requires additional cleaning by passage through a Porapak column^38^, the total preparation time is reduced by a factor of two. Laser spectroscopy and IRMS analysis of Landgrafenbrunnen CO_2_ apparent equilibrium temperatures show values of (10 ± 4) °C and (15 ± 6) °C, respectively, which is in good agreement with the temperature of the well’s water, *T*_w_ = 13.5 °C. A slight deviation from the expected water-CO_2_ equilibrium towards higher temperature has been observed for Stahlbrunnen, *T*_w_ = 12.4 °C versus *T*_IRMS_ = (20 ± 5) °C and *T*_TLDAS_ = (28 ± 7) °C. The interpretation of eventual discrepancies between measured apparent equilibration temperatures and parent water temperatures requires further investigation and is beyond the scope of this paper.

### Future developments

The standard uncertainty of the laser measurements in the 50 ppm range is obtained with samples of about 100 µmol and about 10 sample reference comparisons, which take between 1.5 and 2 h. This is still slightly larger than what can be obtained by mass spectrometry. However, this type of optical measurements is still in its infancy and is expected to improve soon. Already, our 0.8 L Herriott cell can be replaced with a very compact 40 to 140 mL multi-pass cell^39,40^ that provides a similar absorption length. Such small volumes imply reduced sample sizes on the order of 10 μmol or below and lead to faster evacuation times due to much simpler geometry without dead volumes. This shortens the time lapse between sample and reference measurement, thus limiting the impact of instrument drift and reducing the overall measurement uncertainty. We further anticipate that the introduction of an automated pressure balance system will allow for more reproducible conditions that further improve the measurement uncertainty.

It is also worth noting that optical measurements in the *ν*_3_ fundamental region of CO_2_ around 4.4 µm are not exclusively limited to the detection of the ^13^C^16^O^18^O clumped isotopologue. Figure 1 already shows that ^13^C^16^O^17^O can be measured as well. Inspection of spectral data^41,42^ further indicates that the *P*(12)*e* transition at 2305.365327 cm^−1^ provides a well isolated absorption line of ^12^C^18^O_2_. Its reported line strength (*S* = 1.11 ⋅ 10^−23^ cm molecule^−1^) is similar to the intensities of ^12^C^16^O^17^O and ^13^C^16^O_2_ used in this work (Fig. 1) and ^12^C^18^O_2_ should thus well be amenable to quantitative analysis. Given a suitable laser source, the isotopologue can be detected simultaneously with ^12^C^16^O_2_, ^13^C^16^O_2_, ^12^C^16^O^18^O and ^13^C^16^O^18^O and its measurement would provide a second and independent thermometer via the homogeneous

R2 $${}^{12}{\rm{C}}{}^{18}{\rm{O}}_{2}+{}^{12}{\rm{C}}{}^{16}{\rm{O}}_{2}\rightleftharpoons 2{}^{12}{\rm{C}}{}^{16}{\rm{O}}{}^{18}{\rm{O}}$$

exchange reaction, whose equilibrium constant has a temperature coefficient ($d\,\mathrm{ln}\,({K}_{2}/\mathrm{4)/}dT=3.5$ ppm/K) of the same magnitude than *K*_1_ at 300 K (see Fig. 3). The advantage of using this clumped CO_2_ thermometer along with ^13^C^16^O^18^O thermometry is its independence from ^13^C. The presence of kinetic fractionation effects that possibly compromise equilibrium thermometer readings would thus likely be different in the ^13^C containing and in the ^13^C free clumped isotope systems^43–46^. These effects could thus potentially be identified and corrected for. Therefore, optical measurements could provide an entirely new level of temperature information in the future. As an aside, we mention that clumped isotopes are often discussed in terms of bond ordering^4,47^, *i.e*. whether two rare isotopes form a common bond, such as ^13^C-^18^O in ^13^C^16^O^18^O. Reaction R2 is an example of indirect isotope clumping, where the two rare isotopes do not share the same bond^1^. In larger molecules, such as propane, ethane etc. this will be the predominant clumping mechanism. By definition, statistical combination of two different isotopic reservoirs leads to position-independent (anti-)clumping^48,49^. The similar magnitude of isotope fractionation in both reactions R2 and R1 (Fig. 3) demonstrates that thermodynamic clumping effects should also be considered as concentrating two (or more) rare isotopes in the same molecule, leading to a molecular configuration which is thermodynamically more stable than when these isotopes are redistributed over two (or more) different molecules – irrespective whether these isotopes share the same chemical bond or not.

Finally, the direct measurement of the equilibrium constant of an homogeneous exchange reaction at ppm accuracy may provide an interesting benchmark for molecular quantum calculations and potential energy surfaces. At low temperatures, different models are particularly sensitive to ZPE differences (Δ*ν*_0_ and the energies of the lowest states (see Eqs (8) or (9)). At 300 K the ZPE difference factor $\exp (\,-\,{c}_{2}\Delta {\nu }_{0}/T)$ deviates from unity by 5 parts in 10^6^ if Δ*ν*_0_ = 0.001 cm^−1^. This implies that an uncertainty of a few ppm – a range that will be amenable to measurements in the near future – is sufficient to determine ZPE differences at the 0.001 cm^−1^ uncertainty level, irrespective whether the ZPE differences are large, as in the case of the H_2_ + D_2_ $\rightleftharpoons$ 2 HD reaction where Δ*ν*_0_ = 54.867 cm^−1^ ^50,51^, or small – as in reaction R1, where calculated values^22,33,34^ range from 0.433 to 0.435 cm^−1^.

## Summary and Conclusion

We provide the first optical measurement of multiply-substituted isotopologues of CO_2_ at the accuracy level of better than 100 ppm. New advances in laser absorption spectroscopy, such as evidenced by the recent measurement of ^12^C^16^O^17^O at the precision level of 10 ppm within a time frame of 10 min^52^, indicate that laser instruments will favourably compete with mass spectrometer technology very soon. The comparatively high selectivity of laser-based instruments and their large potential of assessing new tracers, such as ^12^C^18^O_2_ for the homogeneous isotope exchange with ^12^C^16^O_2_, will open up new horizons in clumped isotope science and thermometry. The most important advantage of the technology is that the temperature can be obtained easily and directly via an unambiguous measurement of the equilibrium constant of the isotope exchange reaction. The optical CO_2_ isotopologue thermometer is a strong example showcasing the full potential for this and other molecules in the future. We have shown how the technology can be used for the thermometry of gaseous CO_2_ and that new areas related to molecular chemistry and physics are opened up for clumped isotope research. The relatively low cost and size factors of the technology will be additional parameters for developing and easing the spread of this exciting frontier science technology to more laboratories and technological applications.
