# Publisher Correction: Optical clumped isotope thermometry of carbon dioxide

**DOI:** 10.1038/s41598-020-65549-1

**Published:** 2020-06-19

**Authors:** Ivan Prokhorov, Tobias Kluge, Christof Janssen

**Affiliations:** 1 Institute of Environmental Physics, Heidelberg University, 69120 Heidelberg, Germany; 2 Heidelberg Graduate School of Fundamental Physics, Heidelberg University, 69120 Heidelberg, Germany; 3 LERMA-IPSL, Sorbonne Université, CNRS, Observatoire de Paris, PSL Université, 75005 Paris, France

Correction to: *Scientific Reports* 10.1038/s41598-019-40750-z, published online 18 March 2019

The original version of this Article contained errors.

References 6-9 were incorrectly listed as references 11-14. References 10-12 were listed as references 7-9. References 13 and 14 were listed as references 15 and 10 respectively. References 15-16 were listed as references 16-17. References 17-23 were listed as references 19-25. References 24–27 were listed as references 28, 26, 30 and 29 respectively. References 28-33 were listed as references 31-36.

Reference 34 was listed as reference 27. References 35-36 were listed as references 37 and 39 respectively. References 37-40 were listed as references 40-43. Reference 41 was listed as reference 18. References 42-52 were listed as references 44-54. Reference 53 was listed as reference 6. Reference 54 was listed as reference 38.

Furthermore, in the Introduction,

“Typical operation conditions are around *ΔM/M ~* 40000 or lower, which is insufficient to separate ^13^C^16^O_2_ from ^12^C^16^O^17^O at m/z  =  45 or ^13^C^16^O^18^O from ^12^C^17^O^18^O at m/z  =  47, for example.”

now reads:

“Typical operation conditions are around *M/∆M* ~ 40000 or lower, which is insufficient to separate ^13^C^16^O_2_ from ^12^C^16^O^17^O at m/z  =  45 or ^13^C^16^O^18^O from ^12^C^17^O^18^O at m/z  =  47, for example.”

In the lower inset of Figure 1 the two labels P(9) and P(17) were inadvertently switched.

In the Operating Principles and Instrumental Approach section,

“The uncertainty of this calibration is very small: different calculations at 1000 K are given in the literature^22,33,36^ and our calculation based on partition functions evaluated as direct sums of energy levels provided by ab initio calculations that were refined by spectroscopic measurements^34^, indicate that the error at that temperature is about 5 ppm.”

now reads:

“The uncertainty of this calibration is very small: different calculations at 1000 K are given in the literature^22,54^ and our calculation based on partition functions evaluated as direct sums of energy levels provided by ab initio calculations that were refined by spectroscopic measurements^34^, indicate that the error at that temperature is about 5 ppm.”

In Table 2 the column heading,

“CL2012^36^”

now reads:

“CL2012^54^”

Additionally in Table 2 the column heading,

“CBRZ2014^54^”

now reads:

“CBRZ2014^36^”

Finally, the Table 2 legend,

“Different theories are employed: BMU approach using harmonic frequencies for application of the Teller-Redlich^28^ rule and anharmonic correction to the ZPEs – WSE2004^22^. The same approach using harmonic frequencies from another level of theory – CL2012^36^. Path-Integral Monte Carlo (PIMC) evaluation of partition sums – WM2014^33^. Approximate direct sum partition functions from a refined potential surface – CBRZ2014^54^. Direct sum calculation of partition functions (this work) using state energies from new spectroscopic data generated from experimentally refined ab initio calculations^34^. aValues at temperatures other than 200, 300 and 1000 K were recalculated from molecular constants in Table 3 of Wang *et al*.^22^.”

now reads:

“Different theories are employed: BMU approach using harmonic frequencies for application of the Teller-Redlich^28^ rule and anharmonic correction to the ZPEs – WSE2004^22^. The same approach using harmonic frequencies from another level of theory – CL2012^54^. Path-Integral Monte Carlo (PIMC) evaluation of partition sums – WM2014^33^. Approximate direct sum partition functions from a refined potential surface – CBRZ2014^36^. Direct sum calculation of partition functions (this work) using state energies from new spectroscopic data generated from experimentally refined ab initio calculations^34^. aValues at temperatures other than 200, 300 and 1000 K were recalculated from molecular constants in Table 3 of Wang *et al*.^22^.”

These errors have now been corrected in the PDF and HTML versions of the Article.

